# Investigations into the concentration and metabolite profiles of stanozolol and LGD-4033 in blood plasma and seminal fluid using liquid chromatography high-resolution mass spectrometry

**DOI:** 10.1007/s00216-022-04456-y

**Published:** 2022-11-28

**Authors:** Johanna Breuer, Andreas Thomas, Philippe Delahaut, Wilhelm Schänzer, Hans Geyer, Mario Thevis

**Affiliations:** 1 Institute of Biochemistry, Center for Preventive Doping Research, German Sport University Cologne, Am Sportpark Müngersdorf 6, 50933 Cologne, Germany; 2 CER Groupe, Marloie, Belgium; 3 European Monitoring Center for Emerging Doping Agents (EuMoCEDA), Cologne/Bonn, Germany

**Keywords:** Sports doping, Seminal fluid, Stanozolol, LGD-4033, Blood plasma

## Abstract

Potential scenarios as to the origin of minute amounts of banned substances detected in doping control samples have been a much-discussed problem in anti-doping analysis in recent years. One such debated scenario has been the contamination of female athletes’ urine with ejaculate containing doping agents and/or their metabolites. The aim of this work was to obtain complementary information on whether relevant concentration ranges of doping substances are excreted into the ejaculate and which metabolites can be detected in the seminal fluid (sf) and corresponding blood plasma (bp) samples. A method was established to study the concentration and metabolite profiles of stanozolol and LGD-4033—substances listed under anabolic substances (S1) on the World Anti-Doping Agency’s Prohibited List—in bp and sf using liquid chromatography high-resolution mass spectrometry (LC-HRMS). For sf and bp, methods for detecting minute amounts of these substances were developed and tested for specificity, recovery, linearity, precision, and reliability. Subsequently, sf and bp samples from an animal administration study, where a boar orally received stanozolol at 0.33 mg/kg and LGD-4033 at 0.11 mg/kg, were measured. The developed assays proved appropriate for the detection of the target substances in both matrices with detection limits between 10 and 40 pg/mL for the unmetabolized drugs in sf and bp, allowing to estimate the concentration of stanozolol in bp (0.02–0.40 ng/mL) and in sf (0.01–0.25 ng/mL) as well as of LGD-4033 in bp (0.21–2.00 ng/mL) and in sf (0.03–0.68 ng/mL) post-administration. In addition, metabolites resulting from different metabolic pathways were identified in sf and bp, with sf resembling a composite of the metabolic profile of bp and urine.

**Graphical Abstract:**

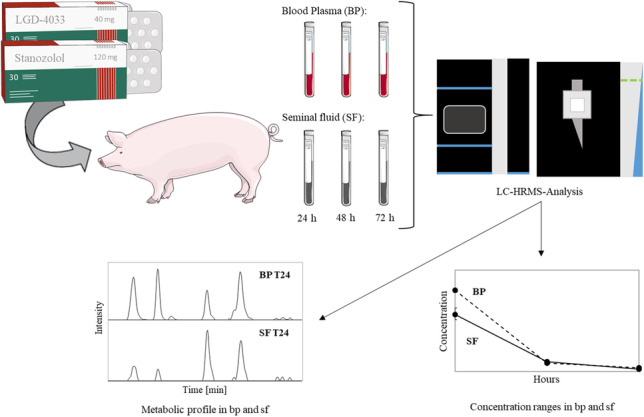

## Introduction

Testing for performance enhancing substances has become an integral part of professional sports. This involves both in-competition and out-of-competition doping controls for prohibited substances and methods of doping, documented in the Prohibited List annually published by the World Anti-Doping Agency (WADA), and frequently (yet at low percentage), doping controls result in adverse analytical findings (AAFs) [1, 2].

Modern analytical tools help to extend the detection windows for prohibited substances in the context of anti-doping, and further research into metabolic biotransformation processes has ensured improving limits of detection (LOD) and the long-term traceability of drugs and their metabolites in blood, urine and other matrices. This generally desirable superior detection capability that is vital for appropriate sports drug testing programs can, however, also lead to AAFs, where unintentional and/or unknown exposure of an athlete to drugs or drug residues occurred. Such cases included for instance the use of contaminated dietary supplements or the exchange of contaminated body fluids [3–6].

The latter represents a specific situation with regard to the possibility of drug and metabolite transmission through intimate contact, i.e., ejaculate/seminal fluid (sf). Here, one conceivable scenario concerns the contamination of a female athlete’s urine sample with drug (metabolite) residues present in the partner’s sf through sexual intercourse; hence, in a first step, it has become necessary to establish means to identify ejaculate in female urine. Recently, semenogelin (sg), a protein specific for human sf, was employed as a marker of sf after sexual intercourse in women’s urine by immunological and chromatographic mass spectrometric methods [7]. The detection of sg in urine alone cannot prove that a prohibited substance or its metabolites entered the urine through ejaculate, but the absence of sg would rule out the scenario of intimate contact as the main reason for an AAF [7].

Further, information on appearance and approximate quantity of prohibited substances (and/or their metabolites) in ejaculate are of great importance for result management when scenarios of potential urine contamination with sf are debated. Here, literature data is scarce, but the summary published by Pichini et al. corroborates the fact that a variety of drugs are present in sf when administered [8]. It is assumed that the concentration of the substances in ejaculate depends on their distribution into the different glands’ fluids composing the ejaculate mixture (prostate 30%, seminal vesicle 60%, epidermis, ampulla, bulbourethal, and urethral glands 10%) [9, 10]. Recently, endogenous hormones (androgens, estrogens, corticoids) and psychotropic drugs such as antidepressants have also been detected in sf [11, 12] Different distribution patterns of drugs between blood and sf were reported, with some compounds exhibiting higher blood concentrations and others were observed at similar levels in both matrices.

To assess the plausibility of AAFs caused by intimate contact in general, information on the approximate concentration ranges of substances commonly abused in sports such as anabolic agents in sf would be helpful [13, 14]. In this study, two different substances from the class of anabolic agents are investigated as to their appearance and concentrations in sf, with LGD-4033 representing a selective androgen receptor modulator (SARM) recently discussed in the context of an AAF related to intimate contact, and stanozolol (Stan) as a typical representative of anabolic steroids [5, 15].

The aim of this work is the development of a model for testing the blood plasma (bp)/sf concentration ratio of substances classified in the WADA Prohibited List as anabolic agents [2]. For this purpose, an animal administration study was conducted with a boar receiving a single oral dose of these compounds. The sf and bp samples were analyzed by liquid chromatography high-resolution mass spectrometry (LC-HRMS) to compare the different concentration levels. In addition, the metabolite patterns in sf and bp were investigated, thus providing an indication whether different metabolic pathways or distributions of the substances might exist.

## Material and methods

### Chemicals and reagents

Reference material for Stan was obtained from Sigma-Aldrich (Darmstadt, Germany). The metabolites 3′-OH-Stan, Stan-N-glucuronide, and 3′-OH-Stan-O-glucuronide  (3'-OH-Stan-Gluc) were obtained from Cerilliant (Round Rock, TX, USA); 4β-OH-Stan, 4α-OH-Stan, 16β-OH-Stan, 16α-OH-Stan, and 17-Epi-16α-OH-Stan were synthesized in-house as previously described [16]. LGD-4033 reference material was obtained from SelleckChem (Houston, TX, USA). As internal standards (ISTD) Stan-d_3_ from LGC Standards GmbH (Wesel, Germany) for Stan and the SARM S-24 for LGD-4033 (synthesized in-house as described previously [17]) was used. Acetonitrile (ACN) was obtained from VWR chemicals (Radnor, PA, USA). Ammonium acetate (NH_4_Ac) and acetic acid (AcOH) were obtained from Merck (Darmstadt, Germany). Formic acid (FA) was obtained from Biosolve (Valkenswaard, Netherlands). Methanol (MeOH) was purchased from J.T.Baker (Phillipsburg, NJ, USA). Ultrapure water was received from a Barnstead GenPure xCAD Plus from Thermo Scientific (Bremen, Germany). Sample collection for blank bp samples from healthy human volunteers was approved by the local ethics committee (139/2021), all participants provided written informed consent, and samples were stored at -20 °C until use.

### Administration study

An administration study employing an adult boar was conducted with a single oral dose of Stan (120 mg, 12 pills Winstrol from Zambon Farmaceutici (Bresso, Italy), each containing 10 mg) and LGD-4033 (40 mg from SelleckChem (Houston, TX, USA), 4 mL of a 10 mg/mL DMSO solution). Bp (5–10 mL) and sf (250–350 mL) were collected in pairs before and 24 h, 48 h, and 72 h after administration. Boar sf was obtained using a dummy sow setup. The bp and sf samples were stored at − 20 °C after collection and centrifugation. The analysis of sf was processed six-fold (*n* = 6), while bp was processed only once due to the substantially more limited volume and conservation for further investigations in a separate project. Additional blank sf samples were collected from ten other boars for method development and validation.

The animal study was performed with the approval of the Belgian Ministry of Small Businesses and Agriculture (LA1800104) and in compliance with Directive 2010/63/EU of the European Parliament and of the Council on the protection of animals used for scientific purposes.

### Sample preparation

To 500 µL of sf or 200 µL of bp, 10 μL of ISTD working solution (5 ng/mL S-24, 10 ng/mL Stan-d_3_) was added using a safe-lock-Eppendorf™ tube (2 mL sf; 1.5 mL bp). To precipitate proteins, 1500 µL (sf) or 600 µL (bp) ice-cold ACN was added and the sample was vortexed for 20 s. Following centrifugation (10 min, 17,000 × *g*) the pellet was discarded. The supernatant was evaporated to dryness in an Eppendorf™ (Hamburg, Germany) Concentrator plus system at 60 °C and 14,000 rpm. The dried sample was reconstituted in 50 μL of 50 mM ammonium acetate buffer (containing 0.1% acetic acid, pH 4.5)/ACN (80/20, v/v).

### LC-HRMS analysis

Two LC-HRMS methods were developed for the analysis of Stan and LGD-4033. The LC-HRMS analysis for LGD-4033 was adapted and modified from Wagener et al. [18]. The LC-HRMS system used was a Vanquish HPLC system coupled to an Orbitrap Exploris 480, both from Thermo Fisher (Bremen, Germany). For chromatography, a Poroshell 120 EC-C18 analytical column (Agilent (Waldbronn, Germany) 50 × 3.0 mm, 2.7 μm particle size) was used connected to an EC 4/3 Nucleoshell RP 18 Plus guard column (4 × 3 mm, 5 μm particle size) from Macherey–Nagel (Düren, Germany). The gradient with a run time of 16.5 min operated with 5 mM NH_4_Ac buffer containing 0.1% acetic acid (pH 4.5) as solvent A and ACN as solvent B. The method was started with 25% B and flow rate of 200 µL/min, increasing to 40% B in 5 min and to 100% B in another 6 min. Subsequently, 100% B was held at 200 µL/min for 1 min and 350 µL/min for 1 min, followed by re-equilibration at 25% B for 3 min at 350 µL/min and 0.5 min at 200 µL/min. The injection volume was 5 µL.

Measurements were made in negative (LGD-4033) or positive (Stan) ionization mode with an ionization voltage of − 3000/ + 3500 V and a transfer tube temperature of 350 °C. A resolution of 60,000 full width at half maximum (FWHM) was selected for the full-scan and HRMS/MS experiments, and the full-scan was performed in a range of *m*/*z* 100–800. The normalized collision energy (NCE) was 30% for all deprotonated molecules of LGD-4033-derived analytes except the analytes with precursor ions at *m*/*z* 353, for which 20% was used. In case of Stan-derived compounds, 72% NCE was employed, except for the protonated precursor molecules at *m*/*z* 345, for which 72% and 45% were utilized. For product ion scan experiments, the isolation window in the quadrupole was set to 1 m/*z*. Nitrogen was generated by the CMC nitrogen generator (Eschborn, Germany) and used as collision gas. The Orbitrap 480 was calibrated regularly with the manufacturer’s calibration solution.

### Assay characterization

The assay was characterized for the intact and unmetabolized compounds Stan and LGD-4033 based on criteria outlined in the WADA International Standard for Laboratories for non-threshold-substances [19]. The assessed assay characteristics included selectivity, reliability, limit of detection (LOD), linearity, accuracy, precision, carryover, recovery, and matrix effects in boar sf and human bp. The selectivity of the method was determined by analyzing ten blank boar sf and ten human bp samples. Additionally, the reliability of the method was confirmed by identifying LGD-4033 and Stan at 1 ng/mL in ten spiked samples of each matrix. The precision was determined by analyzing matrix samples containing Stan and LGD-4033 at 0.1 ng/mL, 0.25 ng/mL and 0.5 ng/mL. LODs were determined using the following detection criteria: the presence of at least two diagnostic ion transitions at the respective retention time of the analyte with a mass error of 10 ppm (or less) and a signal-to-noise ratio of 3:1 determined visually using the ThermoFisher™ QualBrowser™. Carryover was determined by measuring the analyte at 4 ng/mL and probing for the presence of the analytes in subsequently injected blank samples. Recovery was analyzed by spiking six matrix samples each with the respective analytes before and after protein precipitation. The matrix effect was determined by comparing analyte/ISTD ratio abundances in six spiked samples in the LC solvent A and sample matrix, respectively. Further, a calibration curve was prepared with spiked matrix samples at 7 concentration levels (0.02, 0.05, 0.1, 0.2, 0.5, 1, and 2 ng/mL).

## Results

### Assay characterization

The results of the assay characterization for Stan and LGD-4033 are summarized in Table 1. No interfering signals occurred either in the sf samples from ten different boars or in the bp from ten different humans, and the method was considered selective. For the purpose of the study, qualitative detection and calibration curves supporting the estimation of concentration levels of selected analytes within the defined working range was considered sufficient with the assay offering LODs between 10 and 40 pg/mL and demonstrating linear responses over the concentration range of the analytical procedure for sf and human bp. Carryover and precision had acceptable values and were considered appropriate for this application. Recovery and matrix effects were determined, in accordance with WADA guidelines [20], at mid-range concentration levels using spiked blank matrix samples. Porcine/boar blood is often described as similar in quality to human blood and used in an analogous way, although there are differences in composition [21]. For the validation of the method, the qualitative commonality is considered sufficient; a limitation has been though that the validation results only apply to Stan and LGD-4033, and assay characteristics might be transferable to the metabolites only to a limited extent.

**Table 1:** Summary of LC-HRMS/MS validation results for LGD-4033 and Stan

Validation parameter	LGD-4033		Stan		Concentration(s)	Repeats
	Bp	Sf	Bp	Sf		
Selectivity	0\10	0\10	0\10	0\10		*n* = 10
Intra-day precision	5.6%	9.5%	5.4%	6.2%	0.5 ng/mL	*n* = 6
Intra-day precision	5.5%	15.5%	3.6%	8.1%	0.25 ng/mL	*n* = 6
Intra-day precision	7.6%	11.9%	4.2%	5.5%	0.1 ng/mL	*n* = 6
LOD	40	10	20	10	pg/mL	*n* = 10
Reliability	10/10 (6.0%)	10/10 (9.9%)	10/10 (6.0%)	10/10 (7.3%)	1 ng/mL	*n* = 10
Recovery	73.4–80.1%	72.6–80.2%	58.4–68.7%	74.4–82.6%	1 ng/mL	*n* = 6
Matrix effect	82.8–114.6%	65.0–84.0%	75.0–115.8%	84.5–112.2%	1 ng/mL	*n* = 6
Carryover	< 1.0%	< 1.0%	< 1.0%	< 1.0%	4 ng/mL	*n* = 1
Linearity (*R*^2^)	0.9960*	0.9914	0.9988	0.9988	0.02–2 ng/mL	*n* = 6

*0.05–2 ng/mL

### Administration study: Stanozolol

**Fig. 1:**
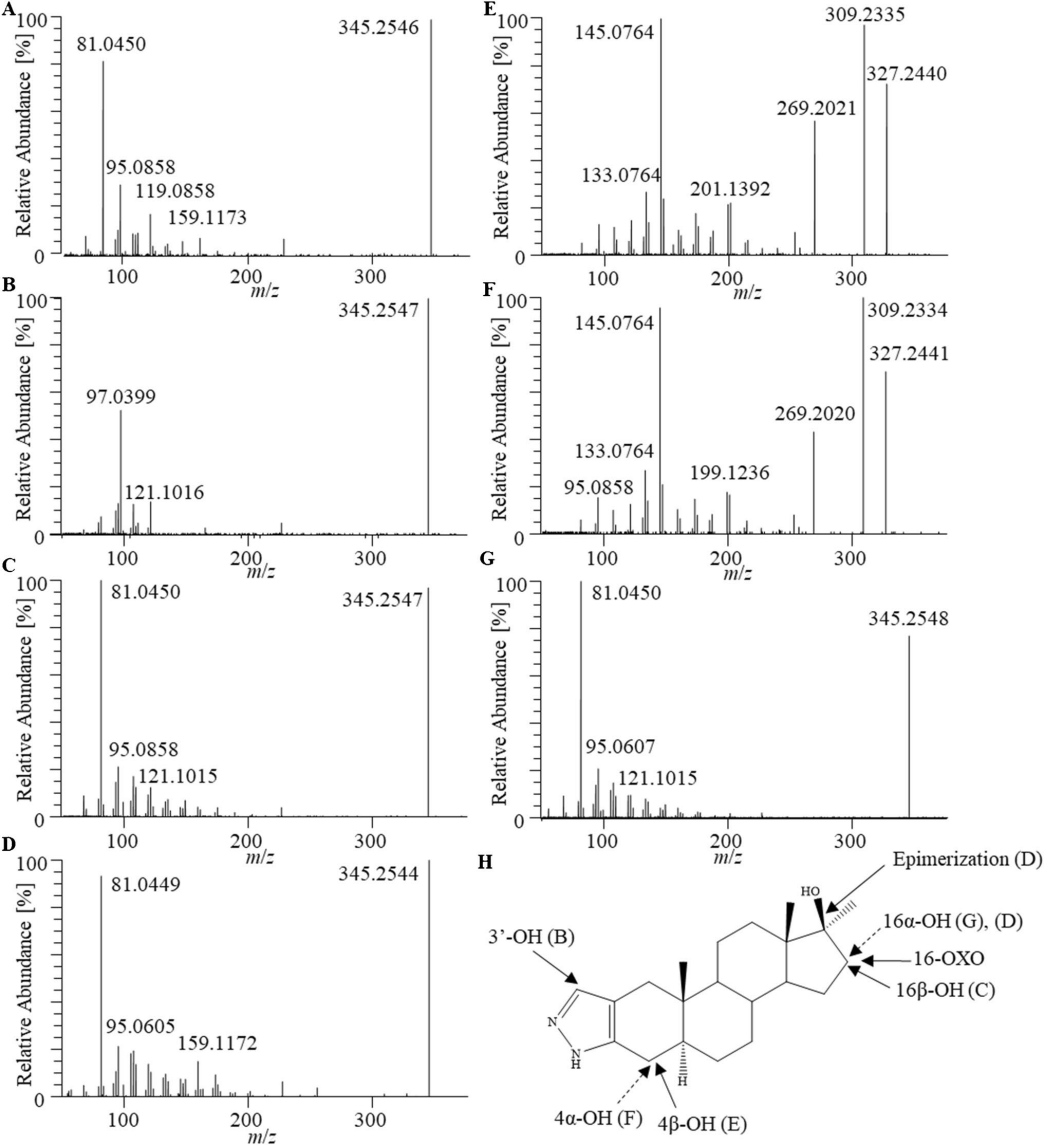
ESI-product ion mass spectra ([M + H].^+^  = 345) of (**A**) unknown hydroxy-Stan metabolite (boar sf sample), (**B**) 3′OH-Stan (reference standard in boar sf), (**C**) 16β-OH-Stan (boar sf sample), (**D**) 17-Epi-16α-OH-Stan (reference standard in boar sf), (**E**) 4β-OH-Stan (reference standard in boar sf), (**F**) 4α-OH-Stan (reference standard in boar sf), (**G**) 16α-OH-Stan (boar sf sample) recorded with a CE of 72% for **A**–**D** and **G**, CE of 45% for **E** and **F**, on an Orbitrap Exploris 480. (**H**) Structure of Stan with arrows indicating metabolic reactions (adapted and modified from Scarth et al. [24])

Reference materials of Stan and metabolites were spiked in blank sf and bp to identify retention time and diagnostic product ions (Table 2). The precursor ion of Stan remains intact to a substantial level even at high collision energies and dissociates into specific product ions such as *m*/*z* 81.0447 that is characteristic for the pyrazol moiety. Consequently, the product ion at *m*/*z* 97.0396 is indicative for an oxygenation in that residue as observed in case of 3′-OH-stan. The product ion at *m*/*z* 145.0760 is typically observed with a hydroxylation of Stan at position C-4 and only detected at lower NCEs (Fig. 1E, F) [22]. The reference standard of 16α-OH-Stan elutes considerably earlier than 16β-OH-Stan. The elution order agrees with the sequences described in the literature, although the therein reported retention time delta was smaller. [23, 24] A possible explanation is the fact that a comparably low LC flow rate (200 µL/min) was chosen in order to optimize the separation of target analytes.

**Table 2:** All metabolites of Stan in bp and/or sf after animal administration or spiking with reference standards. Product ions in bold were used to determine the relative intensity of metabolites and unmetabolized Stan

Analyte	Metabolic transformation	Found in matrix	Precursor ion (*m/z*)	Formula	Retention time (min)	Product ions (*m/z*)
Stan	-	Sf/Bp	329.2598	C_21_H_33_N_2_O^+^	9.44	**81.0447**
						107.0855
S-M1	17-epimerization	Bp			10.32	**81.0447**
						107.0855
S-M2-a	Glucuronidation, hydroxylation	Sf/Bp	521.2857	C_27_H_41_N_2_O_8_^+^	4.50	**345.2537**
						81.0447
3′-OH-Stan-O-Gluc S-M2-b		Sf/Bp			5.45	**345.2537**
						97.0396
S-M2-c		Bp			6.99	**345.2537**
						81.0447
16α-OH-Stan S-M3-a	Hydroxylation	Sf/Bp	345.2537	C_21_H_33_N_2_O_2_^+^	4.98	**81.0447**
						95.0855
S-M3-b		Sf/Bp			6.45	**81.0447**
						95.0855
4α-OH-Stan		/			6.23	**145.076**
						95.0855
3′OH-Stan		/			6.60	**97.0396**
						121.1012
4β-OH-Stan		/			7.00	**145.076**
						95.0855
16β-OH-Stan S-M3-c		Sf/Bp			7.12	**81.0447**
						95.0604
16α-OH-17-epi-Stan		/			8.00	**81.0447**
						95.0604
S-M4-a	Bis-hydroxylation	Bp	361.2486	C_21_H_33_N_2_O_3_^+^	2.24	**81.0447**
						121.4025
S-M4-b		Sf/Bp			4.34	**81.0447**
						123.0789
S-M5	Oxidation	Sf/Bp	343.2380	C_21_H_31_N_2_O_2_^+^	6.93	**81.0447**
						257.2012
S-M6	?	Sf/Bp	359.2329	C_21_H_31_N_2_O_3_^+^	3.23	**81.0447**
						273.1961
S-M7-a	Sulfate, hydroxylation	Bp	425.2105	C_21_H_33_N_2_O_5_S^+^	3.07	**81.0447**
						345.2537
S-M7-b		Sf/Bp			3.35	**81.0447**
						345.2537
S-M7-c		Bp			3.78	**81.0447**
						345.2537
Stan-d_3_ (ISTD)		Sf/Bp	332.2766	C_21_H_30_N_2_OD_3_^+^	9.42	**81.0447**

In all analyzed in vivo post-dose bp and sf samples, non-metabolized Stan was detected. Concentration levels of the drug in both matrices were nearly identical. The semi-quantitatively estimated concentration of Stan after 24 h were 0.4 ng/mL in bp and 0.25 ng/mL in sf, after 48 h 0.04 ng/mL (bp) and 0.05 ng/mL (sf), and after 72 h 0.02 ng/mL (bp) and 0.01–0.02 ng/mL (sf).

**Fig. 2:**
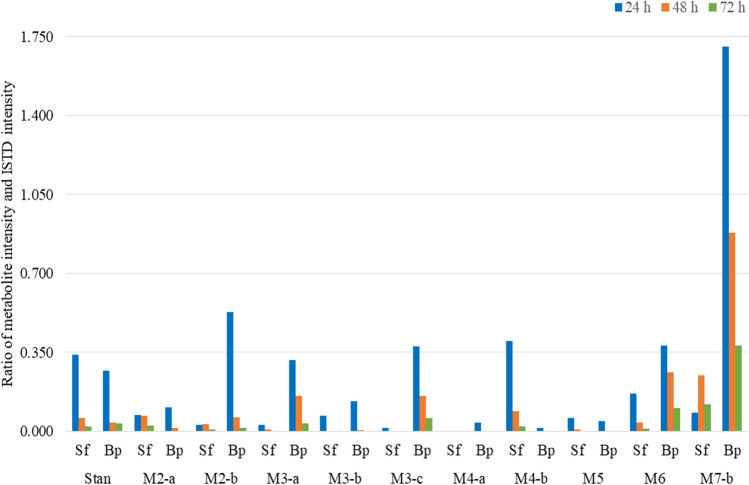
Abundance ratio of Stan and its metabolites normalized to the ISTD after 24 h (blue), 48 h (orange), and 72 h (green) in sf and bp. Metabolites are specified in Table 2

Around twenty phase I and II metabolites from seven different metabolic pathways were detected (Table 2). Of these, four metabolites (3′-OH-Stan, 16α-OH-Stan, 16β-OH-Stan, 3′-OH-Stan-Gluc) could be assigned using reference substances. The signal intensities in the extracted ion chromatograms of most metabolites decreased continuously from the first (24 h) to the last (72 h) post-administration sample and were generally higher in bp than in sf for all metabolites, except S-M4-b (Fig. 2). While the intensity of almost all metabolites was higher in bp than in sf, the concentration and intensity of Stan was similar in both matrices at all three sampling time points.

**Fig. 3:**
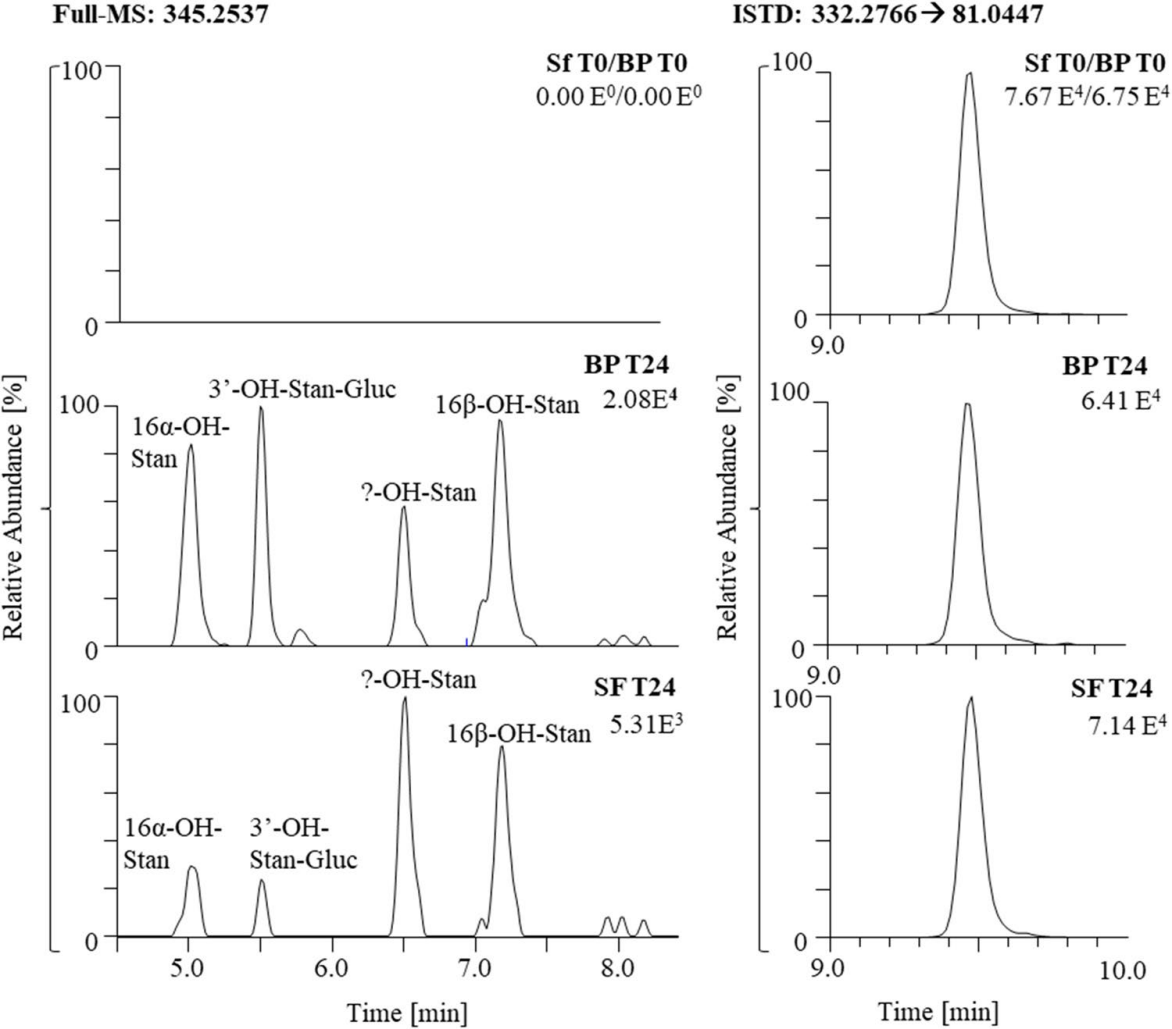
Full-MS extracted ion chromatogram (left) and MS/MS extracted ion chromatogram (right) of sample before administration (combined bp and sf, top) and post-administration sample collected 24 h after application of anabolic agents, indicating the presence of several stanozolol hydroxy metabolites (left) and ISTD (right) in bp (middle) and sf (bottom)

Three hydroxylated metabolites (*m*/*z* 345.2537) were detected at 4.98, 6.45, and 7.12 min, which is shown in Fig. 3 for sf and bp after 24 h. The peak at 5.50 min (Fig. 3) is attributed to in-source dissociation of 3′-OH-Stan-gluc. The metabolite designated as S-M3-a at 4.98 min was assigned to 16α-OH-Stan by reference material and was detected in bp up to 72 h, whereby in sf the analyte was observed only in the 24-h specimen. The metabolite of highest abundance exhibiting a protonated molecule at *m*/*z* 345.2537 after 24 h eluted at 6.45 min (S-M3-b), which could not be assigned to any reference substance. However, hydroxylation at C-3′ or C-4 was excluded since the typical product ions (*m*/*z* 97.0396 or *m*/*z* 145.0760) were not detected in the HRMS/MS spectrum (Fig. 1A). Hydroxylation at C-15, C-6, or another yet unknown site would also be possible as suspected in equine urine samples but without further identification [23, 24]. In greyhound urine, 6α-OH-Stan was determined as the major metabolite after intramuscular injection of Stan [25]. Another peak with product ions consistent with mono-hydroxylation was detected at 7.12 min (S-M3-d, Fig. 3) and was identified as 16β-OH-Stan using a reference standard. This peak had the highest intensity of the hydroxylated metabolites of Stan in bp samples after 24 h and was detectable in all bp samples, whereas in sf such a peak could only be detected after 24 h (Fig. 2).

The metabolites referred to as S-M4 were observed with protonated molecules at *m*/*z* 361.2486, which corresponds to bishydroxylated species of Stan. Two of such metabolites were detected in bp after 24 h (2.24 and 4.34 min), with the latter also being detected in sf. There are several reports where bishydroxy-Stan was detected in human and equine urine [16, 24, 26], but since the signal intensities were low and the product ion mass spectra not sufficiently informative, these metabolites were not further characterized.

**Fig. 4:**
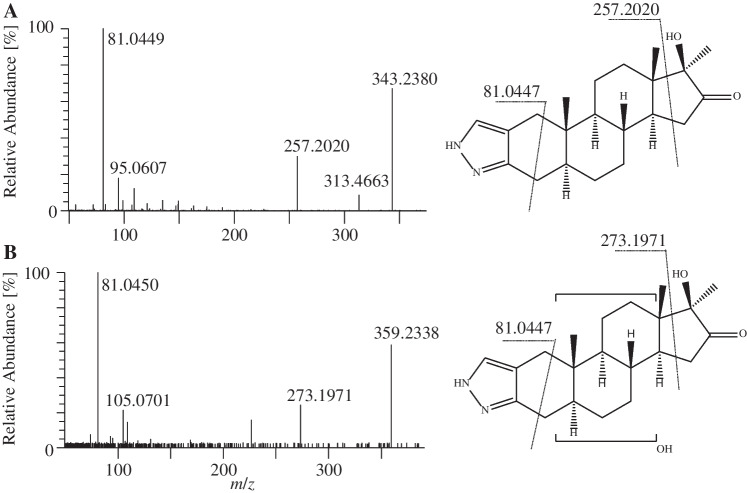
Product ion mass spectra and possible structure of molecules (**A**) S-M5 as 16-oxo-Stan ([M + H]^+^  = 343) and (**B**) S-M6 ([M + H]^+^  = 359)

A metabolite exhibiting a protonated molecule at *m/z* 343.2380, indicating the introduction of an oxo group into Stan, was observed in all post-dose sf and bp samples (S-M5, 24–72 h). The MS/MS spectrum ([M + H]^+^ = 343.2380) shows a product ion at *m/z* 257.2012, which can be attributed to a cleavage of the precursor ion between C-14/C-15 and C-13/C-17 (Fig. 4A), which is consistent with metabolites found and confirmed in human and horse urine that were identified as 16-oxo-Stan with reference standards and in vitro experiments, respectively [24, 27].

One metabolite (S-M6) was determined with an accurate mass and elemental composition corresponding to the introduction of an oxo function and a hydroxyl group. The protonated molecule was found at *m*/*z* 359.2329 in bp and sf samples collected after 24 h only. In consideration of the rationale presented for S-M5, the product ion at *m/z* 273.1971 can also be attributed to the cleavage between C-14/C-15 and C-13/C-17, in combination with a hydroxylation at the steroidal B/C-ring scaffold (Fig. 4B), given the abundant product ion at *m/z* 81.0447 and the lack of a distinct product ion at *m/z* 145.0760. Three different structures are proposed in the literature that could fit a metabolite with these characteristics, i.e., a carboxylation at C-18 or C-20 [26], hydroxy-oxo reaction at C-20 and C-16 [28], and two hydroxylations plus a reduction [29].

The employed LC-HRMS/MS-based method also allowed for the detection of known phase II metabolites [30]. Such phase II metabolites of steroids commonly exhibit an ionization behavior similar to that of the unconjugated analytes [31], which facilitated the detection of three different hydroxylated and glucurono-conjugated metabolites (at *m*/*z* 521.2857) and one hydroxylated and sulfo-conjugated species (at *m*/*z* 425.2105). Two major peaks (4.50 min (S-M2-a), 5.45 min (S-M2-b)) with protonated molecules at *m*/*z* 521.2857 were observed in bp and sf. The S-M2-b was determined by means of reference material to represent 3′-OH-stan-gluc and, based on literature data, S-M2-a was assigned to 16β-OH-Stan glucuronide [27]. The intensity of signals in extracted ion chromatograms was higher in bp than in sf for all glucuronides, and differences in the relative distribution of peak intensities in bp and sf were observed as exemplified with samples collected 24 h post dosing (Fig. 2).

The hydroxylated and sulfo-conjugated metabolite (at *m*/*z* 425.2105, 3.35 min, S-M7-b) was present in all post-administration samples of bp and sf. In bp, two additional signals immediately before (3.07 min) and after (3.78 min) S-M7-b (S-M7-a,-c) with the same accurate mass and similar product ions were detected in the 24 h-specimen, suggesting the formation of further hydroxylated and sulfonated stanozolol metabolites in vivo. In consideration of all available mass spectrometric characteristics, S-M7-b is proposed to exhibit a hydroxyl group at C-16, which is in accordance with observations of Balcells et al., who demonstrated the superior stability of 16-OH-Stan sulfate compared to sulfates of 3′-OH- and 4-OH-stanozolol [32].

### Administration study: LGD-4033

**Fig. 5:**
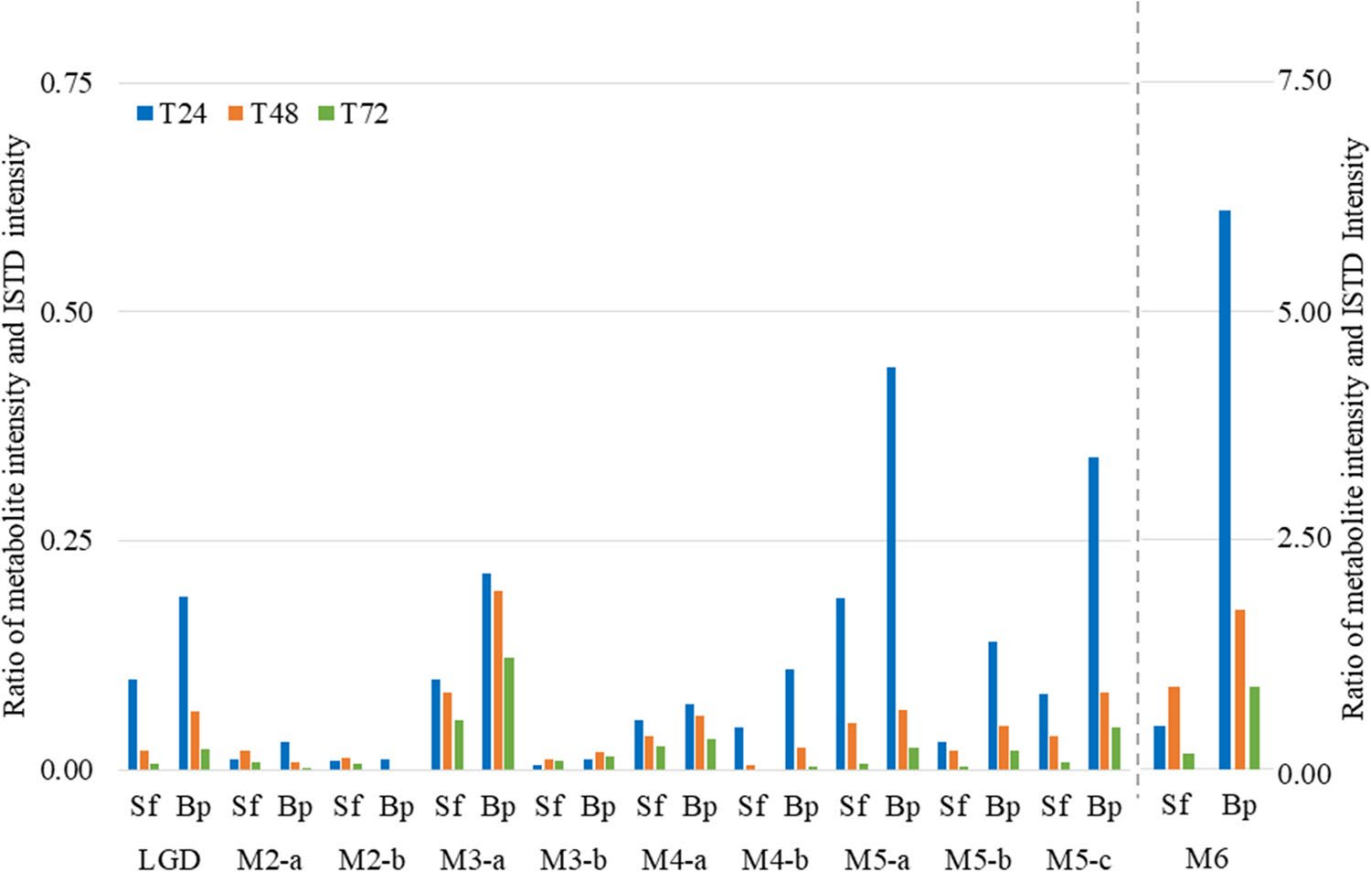
Abundance ratio of LGD-4033 and its metabolites normalized to the ISTD after 24 h (blue), 48 h (orange), and 72 h (green) in sf and bp. Due to the considerable difference in the intensity of the metabolite L-M6 compared to the other metabolites, a secondary axis (right) has been added. Metabolites are specified in Table 3

Fourteen phase I and phase II metabolites from seven different metabolic pathways, which have been thoroughly investigated previously, were detected for LGD-4033 (Table 3) [18, 33, 34]. The intensities of most metabolites of LGD-4033 are higher in bp than in sf, and abundances decrease from 24 to 72 h post-administration for all but one metabolite, which is discussed in more detail (see below, Fig. 5).

**Table 3:** Metabolic transformation, mass/charge ratios of precursor and product ions, sum formulae, and retention times for LGD-4033 and its metabolites detected in bp and/or sf (matrix) in an animal administration study. Product ions in bold were used to determine the relative intensity of metabolites and intact drug

Analyte	Metabolic transformation	Matrix	Precursor ion (*m/z*)	Formula	Retention time (min)	Product ions (*m/z*)
LGD-4033	-	Sf/Bp	337.0781	C_14_H_11_F_6_N_2_O^−^	10.77	**267.0751**
						239.0438
L-M1	Epimerization				11.00	**267.0751**
						239.0438
L-M2-b	Glucuronidation	Sf/Bp	513.1019	C_20_H_19_F_6_N_2_O_8_^−^	7.63	**267.0751**
						337.0794
L-M2-c					7.90	**267.0751**
						337.0794
L-M3-a	Hydroxylation, dehydrogenation	Sf/Bp	351.0574	C_14_H_9_F_6_N_2_O_2_^−^	8.96	**237.0645**
						281.0543
L-M3-b					9.40	**237.0645**
						281.0543
L-M4-a	Hydroxylation	Sf/Bp	353.073	C_14_H_11_F_6_N_2_O_2_^−^	9.49	**255.0751**
						283.0689
L-M4-b					10.13	**283.0689**
						255.0759
L-M5-a	Hydroxylation, ring cleavage	Sf/Bp	355.0887	C_14_H_13_F_6_N_2_O_2_^−^	8.68	**285.0856**
						257.0907
L-M5-b					9.15	**285.0856**
						185.0332
L-M5-c					9.33	**285.0856**
						185.0332
L-M6-b	Bis-hydroxylation	Sf/Bp	369.0679	C_14_H_11_F_6_N_2_O_3_^−^	8.68	**281.0543**
						237.0645
L-M7-a	Tri-hydroxylation	Sf/Bp	385.0628	C_14_H_11_F_6_N_2_O_4_^−^	7.13	**185.0332**
						269.0543
L-M7-b					7.79	**227.0438**
						225.0645
L-M7-c					7.88	**227.0438**
						225.0645
S-24 (ISTD)	-	Sf/Bp	381.0868	C_18_H_13_O_3_N_2_F_4_^−^	10.67	**241.0594**

The most abundant product ion of LGD-4033 under the chosen conditions was *m/z* 267.0751, which was therefore used as quantifier ion. In addition to the identification of LGD-4033 in bf and sf, a second peak (L-M1) eluting shortly after the intact and unmetabolized compound was observed only in the first sample after 24 h and attributed to the LGD-4033 epimer as described by Wagener et al. [18]. The concentration of LGD-4033 was approximately 0.68 ng/mL in sf and 2.0 ng/mL in bp after 24 h, 0.15 ng/mL in sf and 0.63 ng/mL in bp after 48 h, and 0.04 ng/mL in sf and 0.21 ng/mL in bp after 72 h. The intact drug was detected in all post-administration bp and sf samples although, unlike Stan, concentrations were considerably lower in sf than in bp, which supports the assumption that anabolic agents (and their metabolites) as well as other drugs of specific physicochemical nature can partition differently between blood and ejaculate.

Two metabolites referred to as L-M2-a and L-M2-b were identified as glucuronic acid conjugates of LGD-4033 (and its epimer), featuring a deprotonated molecule at *m/z* 513.1019 and the characteristic loss of 176 Da [34]. Unlike for most other metabolites of LGD-4033 in this study, the intensity of L-M2-a and L-M2-b was higher in sf than in bp after 48 h and 72 h (Fig. 5). In a recently published study, the metabolic signatures of the three matrices urine, serum and sf were investigated and compared [35]. Sf was found to resemble the signatures of urine (polar compounds such as glucuronides) and serum (non-polar compounds) which could explain why higher abundances of glucuronides are present in seminal fluid than in blood. Alternatively, minute contaminations of sf with urine in the 48-h sf collection could be considered as glucuronidated metabolites of LGD-4033 predominate in urine. A recent study has shown that human sf can contain small volumes of urine, which is why it cannot be excluded that a fraction of the observed analytes originates from this source [36]; however, considering the difference in average volumes of human und porcine ejaculate, the percentage of urine potentially introducing metabolites into ejaculate in the present study is presumably low.

**Fig. 6:**
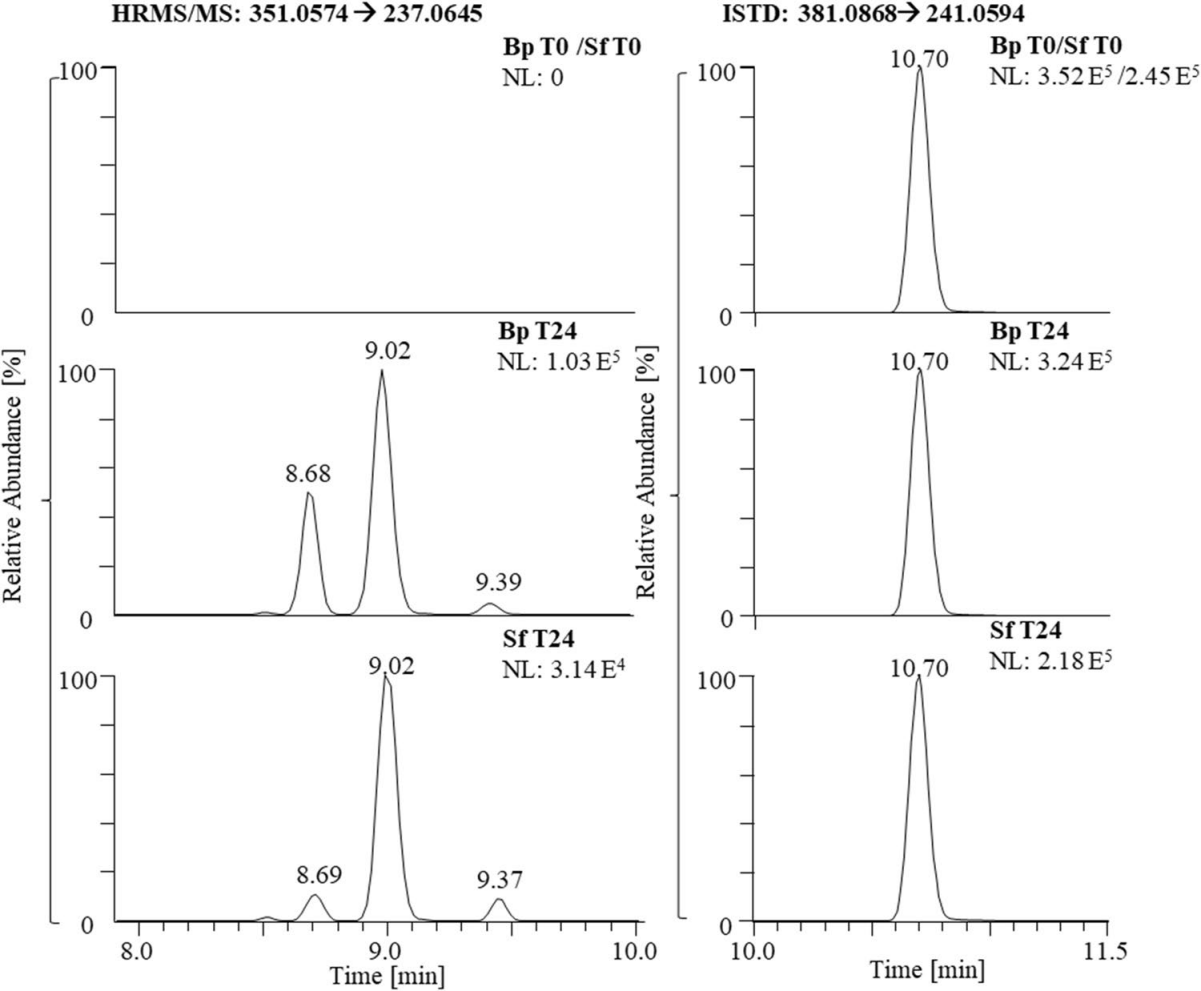
Extracted ion chromatograms of a sample before LGD-4033 administration (combined bp and sf, top) and post-administration samples collected 24 h after application, indicating the presence of three LGD-4033 metabolites (L-M3) in bp (middle) and sf (bottom)

The two metabolites L-M3-a and L-M3-b are related to a deprotonated species at *m/z* 351.0574, which indicates hydroxylation and dehydrogenation (i.e., formation of an oxo-metabolite), which was confirmed by in-house synthesized reference material [18]. A third signal in the extracted ion chromatogram with *m/z* 351.0574 is observed at the RT of bishydroxylated LGD-4033 and has been attributed to an in-source fragmentation rather than a factual metabolite (Fig. 6). The decrease in the intensities of L-M3-a from 24 to 72 h is slow compared to the other metabolites of LGD-4033 and, consequently, its intensities are relatively high after 72 h (Fig. 5). Hence, this metabolite might be suitable to detect LGD-4033 also for a prolonged period of time, which however necessitates further studies including more animals and longer sampling times.

The deprotonated molecule [M-H] ^−^ of the metabolites termed L-M4-a and L-M4-b was found at *m*/*z* 353.0730, indicating mono-hydroxylation of LGD-4033. These metabolites were detected in all post-administration bp and sf samples, and the diagnostic product ion at *m*/*z* 185.0332 localizes the hydroxylation at the pyrrolidine moiety, in agreement with earlier publications [18, 33, 34, 37].

Three metabolites (L-M5-a, -b, -c) exhibiting an [M-H]^−^ at *m*/*z* 355.0887 matched previously reported metabolic products obtained via hydroxylation and opening of the pyrrolidine ring [18, 33, 37]. The retention time of L-M5-a (Table 3, 8.68 min) is, under the employed chromatographic conditions, identical to that of the bishydroxylated metabolite L-M6 (Table 3, 8.68 min), but here a potential in-source dissociation mimicking a metabolite formation is unlikely.

The metabolite L-M6, assigned to bishydroxylated LGD-4033 with the determined *m*/*z* 369.0679, was detected at highest relative abundance of all metabolites in post-administration bp and sf samples. While the intensity of most metabolites decreased continuously from the first to the last sample in both matrices, the intensity of L-M6 peaked with the second sample (48 h) before decreasing at 72 h (Fig. 5). Analyses of human urine of LGD-4033 administration studies were re-analyzed with this method, i.e., without hydrolysis, demonstrating that unconjugated trihydroxylated and bishydroxylated metabolites of LGD-4033 are present in urine (data not shown) [18]. Therefore, contamination with urine (even if at lowest amounts) might contribute to analyte abundances as mentioned above, and thus participate in the increase of L-M6 in the 48-h sample. Hence, in future studies, the collection of corresponding urine samples in addition to bp and sf is advisable for a more complete to better understand metabolite distribution.

The metabolites L-M7-a,-b,-c (*m/z* 385.0625) are assigned to threefold hydroxylated LGD-4033. The intensities of the L-M7 metabolites in bp and sf were low compared to other metabolic products. In the 24-h samples, the intensities of L-M7-a,-b,-c were higher in bp than in sf, but the 48-h samples exhibited higher intensities of L-M7-a,-b,-c in sf.

## Discussion

Banned substances and their metabolites can be present in sf at low or sub ng/mL concentrations and, through intimate contact, scenarios were such sf can be introduced in female urine have been hypothesized and proven in the past [7]. Further to literature data [8], the herein presented proof-of-concept animal experiment provides first insights into concentration ranges attributable to anabolic agents in sf, with ca. 0.25 ng/mL for Stan and 0.68 ng/mL for LGD-4033. Also, data on metabolite patterns and partitioning of drug and drug metabolites between blood plasma and seminal fluid were obtained, complementing the scarce literature data on anabolic agents in ejaculate and the potential transfer of these substances by sexual intercourse, either in a ‘flush-out’ scenario with sf being introduced into a female athlete’s doping control urine sample or by vaginal absorption and subsequent ‘secondary elimination’ into urine. The latter scenario might be less likely considering the herein observed drug and drug metabolite concentration ranges; however, vaginal absorption of therapeutics is an established phenomenon described in the literature and, thus, considering this (presumably remote) possibility, appears warranted [9]. In either case, knowledge about concentration ranges and the extent of metabolites in sf is of utmost importance, and the principal capability of the animal model and analytical approach utilized in this study in providing critical information was demonstrated. However, limitations such as the considerable differences in volumes of seminal fluid between humans and boars might necessitate consideration, and also a potentially species-specific composition of ejaculate and/or routes of metabolism of drugs will require further investigations.

Yet, assuming the herein observed concentrations of stanozolol and LGD-4033 exist also in human sf when commonly reported dosages of the anabolic agents are orally administered, further parameters to factor-in when arguing a potential contamination scenario are the required doping control urine volume of 90 mL and the analytical assay’s LODs. If the analytical assay employed in routine anti-doping does allow for an LOD of 3–7 pg/mL for the anabolic substances in urine, the introduction of 1 mL of sf contaminated with the substances in the concentration range of the animal study into a 90-mL doping control sample could theoretically lead to an AAF. How (repeated) resorption of anabolic agents and their metabolites might contribute to urinary concentrations remains largely unknown, but further dilution compared to sf concentrations is plausible.

## Conclusion

To the best of our knowledge, no data exist on the presence of Stan and LGD-4033 and corresponding metabolites in ejaculate, while metabolic profiles of stan and LGD-4033 reported in other matrices were available and utilized for comparison of metabolites detected in the studied seminal fluid samples. In this exploratory investigation, the banned substances and metabolic products were identified in animal sf, suggesting that a contamination scenario of female (doping control) urine with ejaculate and, hence, drug residues after sexual intercourse cannot be excluded. However, in the animal administration study, the analyte concentrations observed in sf were particularly low, and the plausibility of a contamination scenario considering the parameters of urine volume, potential volumetric load of ejaculate, and urinary drug (or metabolite) concentrations needs to be assessed at the result management level. The obtained results demonstrate that contamination scenarios including drug (metabolite) transfers via seminal fluid are likely to produce AAFs only at low(est) concentration levels. The substantially enhanced analytical sensitivity and required retrospectivity however allow for the detection of such analyte concentrations, and studies such as the herein presented investigation can support assessing the plausibility of case-related arguments.

For future projects, it would be beneficial to collect sf and bp samples over a longer period of time to more accurately assess the duration of detectability of metabolites in sf, as some metabolites were still traceable after 72 h. Metabolites eliminated over a prolonged period of time are of particular interest in anti-doping research for the desired long-term detection of AAS abuse, and for some exogenous AAS (including stanozolol), glucuronide and sulfate metabolites were described to offer particularly long analytical retrospectivity [38] Likewise, in this study, the phase II metabolites appear to offer superior detection windows than the corresponding phase I metabolites. Interestingly, the distribution of Stan and LGD-4033 appeared to be different in bp compared to sf. Hence, further studies investigating different substances are needed to further furnish the knowledge of matrix distribution and elimination profiles especially concerning sf.

## Data Availability

The datasets generated and analyzed as part of this study are available upon request from the corresponding author.
